# Citizen sociolinguistics: A new method to understand fat talk

**DOI:** 10.1371/journal.pone.0217618

**Published:** 2019-05-29

**Authors:** Gina Agostini, Cindi SturtzSreetharan, Amber Wutich, Deborah Williams, Alexandra Brewis

**Affiliations:** 1 School of Human Evolution and Social Change, Arizona State University, Tempe, Arizona, United States of America; 2 College of Dental Medicine, Midwestern University, Glendale, Arizona, United States of America; 3 School of Nutrition and Health Promotion, College of Health Solutions, Arizona State University, Phoenix, Arizona, United States of America; The University of Memphis, UNITED STATES

## Abstract

**Fat talk and citizen science:**

Fat talk is a spontaneous verbal interaction in which interlocutors make self-disparaging comments about the body, usually as a request for assessment. Fat talk often reflects concerns about the self that stem from broader sociocultural factors. It is therefore an important target for sociocultural linguistics. However, real-time studies of fat talk are uncommon due to the resource and time burdens required to capture these fleeting utterances. This limits the scope of data produced using standard sociolinguistic methods. Citizen science may alleviate these burdens by producing a scale of social observation not afforded via traditional methods. Here we present a proof-of-concept for a novel methodology, citizen sociolinguistics. This research approach involves collaborations with citizen researchers to capture forms of conversational data that are typically inaccessible, including fat talk.

**Aims and outcomes:**

This study had two primary aims. Aim 1 focused on scientific output, testing a novel research strategy wherein citizen sociolinguists captured fat talk data in a diverse metropolitan region (Southwestern United States). Results confirm that citizen sociolinguistic research teams captured forms of fat talk that mirrored the scripted responses previously reported. However, they also capture unique forms of fat talk, likely due to greater diversity in sample and sampling environments. Aim 2 focused on the method itself via reflective exercises shared by the citizen sociolinguists throughout the project. In addition to confirming that the citizen sociolinguistic method produces reliable, scientifically valid data, we contend that citizen sociolinguist inclusion has broader scientific benefits which include applied scientific training, fostering sustained relationships between professional researchers and the public, and producing novel, meaningful scientific output that advances professional discourse.

## Introduction

Research projects that entail collaborations with lay people (citizen scientists) are common in the natural and biological sciences where citizen science has made an enormous and positive contribution to the scaling of data collection efforts [1]. This is especially true when it comes to studies focused on complex or abstract data whose documentation involves advanced reasoning or inferential skills that cannot be reliably outsourced to technology [2]. Abstract data arguably comprise the bulk of that seen in social science research, and yet the adoption of citizen science as a research strategy remains conspicuously absent here [3]. Within the social sciences, some types of linguistic data are spontaneously situational and therefore difficult to capture in systematic fashion (e.g., complaints, apologies, terms of address/reference, greetings, and so on). Collaborations of linguists and other citizen scientists in this domain could help capture greater diversity in factors including scale, location, network, social interaction, and sociocultural variables than is typically seen in the current literature. As stated by Dickinson et al. [4:291] in the context of ecological research, “[d]ispersed data collection and the ability to collect observations and connect with people, in places, and at scales that would otherwise not be possible render citizen science increasingly important.” This is particularly true given the widespread use of smart devices to facilitate standardized, large-scale data collection efforts by researchers *in situ* and in real time [3, 5]. The relative paucity of citizen *social* scientists (and associated loss of scientific knowledge) has been noted by several scholars, including linguists Rymes and Leone [6], who have championed for the creation of citizen sociolinguistics, specifically.

The definition of citizen sociolinguistics remains unsettled. While the inclusion of laypeople in research projects is one obvious aspect of such work, opinions differ as to the nature of their involvement. Svendsen [7] states this burgeoning field is defined by the engagement of lay people in *the process* of sociolinguistic research. Fitting with the approach we apply here, this can range from data documentation to more complex tasks associated with analysis and interpretation. Rymes and Leone [6], however, define citizen sociolinguistics using a more metalinguistic lens, focusing on the secondary analysis of language by everyday people as they reflect publicly (e.g., via social media) on language phenomena (e.g., use of accent/dialect, non-prescriptive grammar forms, monolingualism, etc.). In effect, the layperson becomes the *citizen sociolinguist* and analyst at the moment they make comments on everyday language uses. Rymes et al. [8] therefore seem to suggest that the citizen sociolinguist need not be included in the execution of research (or even be aware of it).

Despite these differences in perspective on the defining boundaries of practice, both Rymes’ et al. and Svendsen’s approaches entail some form of indirect or direct engagement with local citizens to understand otherwise inaccessible language data. Both would also appreciate that citizen sociolinguists may often be better situated than professional linguists to understand the relevance of language data heard in everyday environs. Moreover, both perspectives contend (albeit to variable degrees) that citizen sociolinguistics could have a democratizing effect on the acquisition of linguistic data by dismantling the artificial boundary between the researcher and researched, facilitating bidirectional dialogue between the two and changing the “traditional way we conceive science and who has the authority to do it” [7:3]. Certainly, the study of language has historical roots in community-based research [9–12], but citizen sociolinguistics can expand on this tradition in a distinct way. Engaging local field-site residents, community members, and students in language studies is common practice in the social sciences [10, 12–14] where people are selected to participate based on their willingness in combination with other characteristics or skills (e.g., being identified lay experts of the language form under investigation). In an era where travel, relocation, and online interaction are the norm [15–16], citizen sociolinguists may increasingly provide a means of critical research support by virtue of the fact they can capture data while navigating multiple daily social networks—networks in which interactions and the relationships that shape them are multimodal and complex [17–18], and for which sociolinguistic data may be more unconscious than that produced via focus groups, prompts, or surveys (what [3] termed *hidden data*). Citizen sociolinguistics therefore enriches the research goals by scaling-up the project, flattening power asymmetries, and capturing a more diverse array of speakers and speaking styles.

Here we report on the methodological outcomes of a novel citizen sociolinguistics project designed to leverage citizen social scientists to identify and systematically capture spontaneous, verbal interactions, a key form of linguistic data. We follow Svendsen [7] in our interpretation of citizen sociolinguistics in that we include lay people in the actual *doing* of research. Volunteers from the local community were trained to be citizen sociolinguists and were embedded in research teams, identifying and documenting a specific conversational interaction called “fat talk.” Fat talk consists of verbal exchanges wherein speakers disparage their own bodies; the disparagement often takes the form of a request for evaluation from fellow interlocutors [19]. For example, fat talk frequently includes self-deprecating or body-checking utterances like “I look so fat!” or “Does this make me look fat?” which typically evoke a scripted conversational response (such as “No, you don’t”) [19–21]. The response to fat talk seems to depend on the perceived status, identity, familiarity, and cultural backgrounds among interlocutors. Therefore, fat talk is sociolinguistically important precisely *because* it is rooted in interaction, making audible the commonly held, deep-seated concerns about physical appearance shaped by broader sociocultural norms [20–21].

Fat talk was first identified by Nichter and Vukovich [19] who investigated the pervasiveness of diet-focused culture in American adolescents (see also [20–21]). Self-deprecating or self-evaluating statements such as “My thighs are so fat!” or “Do these jeans make me look fat?!” were frequently expressed by the young people in Nichter’s focus groups [20]. Since then, expanded investigations of fat talk have confirmed it to be common among women of all ages [22–23], and indeed the focus of most fat talk studies has been on women (e.g., [24–25]). However, reports of fat talk among men has increased in recent years ([21, 26–27]), particularly in reference to body shame and weight stigma (e.g., [28–30]). While there is some variation in the data collection tools used in these studies, most utilize structured means of data collection that include self-report/recall exercises, lab-based experiments with confederates, discourse completion tasks and, as in the case of Becker et al [31], a free list response to a fat talk prompt. While these reveal important dimensions about fat talk cultural scripts, their structured nature masks whether these scripts are evident in fat talk when it arises organically and spontaneously in different social interactions.

While outwardly mundane on its surface, fat talk can have potentially important impacts on interlocutors beyond its interactional significance. At the group level, there may be some positive outcomes, such as greater group cohesion and an inclusive recognition of broader social norms [20]. However, outcomes appear largely negative at the individual level, and can increase negative health effects including body dissatisfaction and disordered eating [32–36]. Despite this clear connection to psychosocial health, fat talk has remained virtually undocumented in its naturally-occurring forms precisely because it is typically expressed in ways that are brief and spontaneous, being commonly woven into or in between other conversations (much like small talk) [37]. For this reason, most prior academic work on fat talk relies upon written prompts in which participants are asked to respond to artificial interactions or to listen to a fat talk statement and then complete a body dissatisfaction survey [25,38]. Fat talk was an ideal candidate for this nascent study in citizen sociolinguistics for multiple reasons, both in terms of advancing sociolinguistic understanding on this understudied topic and in terms of citizen sociolinguist engagement. These include its status as a verbal interaction shaped by sociocultural norms, its clear connection to psychosocial health and group cohesion, the brief nature of its expression being an accessible data target for new citizen science researchers, and the public’s general fascination with this form of speech (see [39–41]).

One consistent theme across researchers who look at citizen science with a critical eye is that it should do more to democratize the process, specifically that it should alter “the relation between the researcher and the researched” [7:139, 42] and increase the scope of practitioners who have the authority to “do science.” At a basic level, this requires that such projects go beyond seeing the citizen scientist as a “mere” data collection assistant [43–46] and instead create opportunities for meaningful inclusion and sustained dialogue between academics and lay folk. Thus, an additional challenge we undertook in this work was to ensure that citizen sociolinguists were integrated into the interpretation and analysis of sociolinguistic data. In this way, our project is designed to assess whether citizen sociolinguistics can simultaneously increase public knowledge while advancing scientific process and understanding in novel ways [6–7].

The primary aim of this project was to test the feasibility of citizen sociolinguistic methods for capturing large numbers of public fat talk utterances. This entailed assessing fat talk captured by citizen sociolinguistic research teams for its adherence to already understood forms of fat talk reported in the literature. We assess the methodological outcomes of our work in three domains: (1) locational diversity of data collected, (2) gender bias in data collection, (3) age bias in data collection. In each case, we compare the diversity of data collected by a citizen sociolinguistic method to that typically reported in the literature to determine if the former can significantly widen our understanding of fat talk. Another aim was to assess efficacy of citizen sociolinguistics in creating meaningful inclusion and dialogue between academics and lay people. As such, we assessed citizen sociolinguists’ (4a) participation in the scientific process and (4b) willingness and ability to act as informal science educators to promote the study’s findings. While detailed data analysis was beyond the scope of this methodology-focused paper (see [47]), we conclude with a brief nod to the sociolinguistic data produced by citizen sociolinguistic research teams (hereafter ‘research teams’), highlighting the ways these contextually rich observations can expand our understanding of fat talk from its primarily psychosocial implications into a solidly sociolinguistic domain.

## Materials and methods

### Recruitment and retention

For this pilot study, ten citizen sociolinguists, 6 women and 4 men ranging in age from 23–60 (mean = 43.4) years old, were recruited in-person from the research team’s networks, primarily from work, religious, community or personal networks (see Table 1). There was wide variation in employment and educational background, and none had any prior training in social science methods. The citizen science literature notes that high attrition is a common problem across citizen science projects [48]. To address this, our research design required routine, sustained interaction between the citizen sociolinguists and the academic research team. This was done to ensure that citizen sociolinguists had some control over the research as well as feeling involved in the project and its execution [49]. Raddick [45] demonstrated that citizen scientists report higher motivation when they personally identify with the project goals or content. Over the six-week course of this study, only one citizen sociolinguist withdrew. As described below, these elements of success–feeling integrated into and in control of the project–were explicitly built into our research design.

**Table 1:** Citizen sociolinguists (CITSLX) profiles.

Participant ID	Gender	Age
CITSLX01	Male	23
CITSLX02	Female	57
CITSLX03	Male	43
CITSLX04	Female	50
CITSLX05	Male	29
CITSLX06	Female	38
CITSLX07	Female	51
CITSLX08	Male	60
CITSLX09	Female	58
CITSLX10	Female	25

### Training

Each citizen sociolinguist was incorporated into an existing team that included one team leader and 2–3 additional researchers. All researchers had formal training in the theoretical and social significance of fat talk and how to identify and document it. Citizen sociolinguists received instruction about the project scope, rationale, protocols, and methods, including how to use a standard template for systematic data collection (see S1 File).

A major method used in this study was participant-observation in which an observer collects data via participation in the day-to-day activities of those being studied. Here it was critical that citizen sociolinguists were both participant and researcher, simultaneously recognizing and documenting target fat talk data. Therefore, citizen sociolinguists were trained in ethnographic methods and documentation for collecting spontaneous instances of publicly expressed fat talk. In doing participant-observation, research teams documented observation locations, information about the participants and bystanders which included simple demographics; information about the successes, failures, and general reflections of the overall research event were logged by the research team as a whole.

Standardized documentation and field demonstrations of data collection improve data integrity and strengthen outsider perceptions of collaborative projects, particularly for multi-site, multi-participant research [50–51]. Therefore, both practices were integrated into our training program and project design. Citizen sociolinguist training consisted of several elements: (1) presentation of fat talk and its relevance, (2) sample phrases of fat talk previously captured by researchers during methods piloting, (3) protocols on how to immediately transcribe overheard utterances onto a paper template, and (4) protocols on how to complete the associated demographic information about interlocutors. In addition to discussing these elements ahead of time, each citizen sociolinguist participated in short-term data collection with his/her research team in the field. Over the course of the research project, they worked collaboratively to document naturally arising fat talk in public spaces to ensure data integrity, accuracy of identification, and consistency in documentation across researchers.

### Team cohesion and support

To ensure project integrity and to move beyond treatment of citizen sociolinguists solely as a means of data collection, all citizen sociolinguists were asked to attend weekly meetings with a research teammate. These meetings were planned at times and locations convenient to the citizen sociolinguists. During these meetings, they completed a brief questionnaire assessing their comfort with the data collection (increased/decreased comfort) and awareness of fat talk (heightened/decreased). Meetings also allowed time to discuss any patterns or themes they noticed in the fat talk and relay any new or ongoing concerns so that project protocols could be updated and shared across all teams as necessary. These weekly check-ins were critical to encourage team cohesion and citizen sociolinguist sustainability, as well as troubleshoot emerging problems. Team leaders submitted the completed questionnaire and a brief summary of the meeting to the study directors for review. Study directors and researchers met weekly to address any problems identified by their respective teams. Citizen sociolinguists also received contact information (e-mail and telephone) for their team leader and study directors to use at any time.

### Data collection

Citizen sociolinguists and their research team members were issued small binders containing standardized, short templates (see S1 File) onto which they transcribed overheard utterances, brief information about the nature of the utterances, site/location, and simple demographic approximations (e.g., ascribed gender, ascribed age, relationship among interlocutors). Citizen sociolinguists were instructed not to audio-record any conversations; they were instructed to be attentive to language occurring around them that was *easily audible* (did not require extra effort to hear and document). Citizen sociolinguists were asked to capture up to 4 utterances (2 turns per speaker) of a relevant conversation. We capped the maximum number of total utterances to 4, allowing the initial utterance of fat talk to be answered, (potentially) restated, and then answered again. Previous scholarship indicates that fat talk is not a sustained interactional genre [37] and people will quickly tire of the exchange (see [52]). At weekly check-ins, citizen sociolinguists would submit completed data sheets to their teammate who was then responsible for digitizing the information; to ensure consistency in transferring written information to digital data, one person per team was responsible for the digitization of all collected fat talk. This entailed transcribing the documented fat talk utterances and accompanying demographic details into a predesigned spreadsheet. Use of the spreadsheet ensured consistent data entry across all research teams and presented a formalized way to conduct subsequent data quality control checks (approximately twenty-five percent of the entire sample underwent quality control checking). This project was subject to independent review by the Arizona State University Office of Research Integrity and Assurance and was awarded exempt status indicating there was no perceived likelihood of harm to human subjects or to participants. Therefore, signed consent was not required.

### Post project reflection

In addition to the weekly check-ins, citizen sociolinguists were also asked to share feedback via a short, post-project questionnaire containing both Likert-type scales and open-ended questions [53]. This allowed participants to reflect on and share their experiences as a citizen sociolinguist after data collection finished. It was distributed in hard copy by teammates at the final project check-in. Nine of ten citizen sociolinguists completed the questionnaire. The purpose of the questionnaire was two-fold: (1) to assess the citizen sociolinguist’s familiarity with the research focus (fat talk) and confidence they had in their own skills related to fat talk documentation, and (2) to solicit information about motivation for participation in the project, perceived benefits/drawbacks of participation, evaluation of project execution, recommendations for future projects, and openness to participating in another citizen science project.

## Results and discussion

### Overview of data collected

Over the course of 6 weeks, over 500 observations were documented by the research teams; after removing those observations which did not qualify as fat talk, 494 unique observations were digitized and prepared for analysis (see S2 File). Of these, 375 (76%) made specific reference to physical appearance at some point during the interaction, and 201 entailed some form of request for body assessment, a critical feature of classic fat talk as described by Nichter [20]. Below we include some examples of observed fat talk documented by the research teams.

Example 1: CSP_FT_0278, two women;
Woman 1 (18–30 years old): I feel like you can see my back fat, can you?
Woman 2 (over 50 years old): I do not see that.

Example 2: CSP_FT_0296, two women;
Woman 1 (18–30 years old): Do my thighs look big in these pants?
Woman 2 (30–50 years old): No way! Big thick thighs are sexy.

Example 3: CSP_FT_0253, two men;
Man 1 (18–30 years old): I’m fat.
Man 2 (18–30 years old): LOL, You’re serious man?

In Examples 1 and 2, the fat talk is phrased as a question by the first speaker and the interlocutor responds to the fat talk with a reply that answers the question. In each of these cases, the reply negates the premise of the fat talk by saying some form of “no.” In Example 3, the fat talk is phrased as a complaint about the self which is heard as a request for evaluation [54–55]; the interlocutor responds first with text-speak “LOL” (‘laugh out loud’) and then follows with a request for clarification or expansion (“You’re serious man?”). In all three cases, fat talk consists of either making a derogatory statement about one’s own body or asking for evaluation or assessment of body size/weight. In each case, the reply is to deny that the speaker looks fat or, in the case of men, to laugh and ask if the speaker is ‘serious,’ thus positioning the very question as ridiculous to begin with, a form of negation of the statement [56]. These examples are typical of the fat talk observed and documented by the research teams [see 47].

### Assessment of data collected by citizen sociolinguists

#### Citizen sociolinguistics data show fat talk occurs in many locations

Of 374 conversations invoking physical appearance, 326 included location information. Results show that fat talk occurs among individuals in many day-to-day public interactions across many different locations. Data collected by research teams show remarkable diversity in the locations where people engage in such talk, ranging from spaces where more personal interactions are likely (e.g., residence communities) to more formal locations (professional offices) to highly populated public venues (parks and sporting arenas or holiday gatherings) (see Table 2). Research team members collected data in all of these various locations. Of these many public settings, over 20% of all interactions were collected from restaurants, bars, and cafes. Interactions from residential locations (e.g., neighborhood spaces) were also common (17%) as were interactions from malls, stores, and shopping centers (16%). These locations are far more diverse than those included in most conventional studies of fat talk, which typically use lab or interview settings [12,25]. In short, it appears that fat talk is pervasive in day-to-day life and is an acceptable topic of conversation across many different contexts.

**Table 2:** Top 5 locations where appearance-related talk was documented.

Location Type	Frequency (%)
Restaurant	21
Residence	17
Mall/Store/Shopping Center	16
University Campus	6
Other	6

While intuitive on its surface, the ways in which location affects fat talk have not been thoroughly investigated in prior sociolinguistic studies. This topic is particularly important given that different forms of fat talk highlight anxieties about the body and are associated with specific psychosocial disorders, as seen in the association of “muscle talk” with muscle dysmorphia or fat talk with disordered eating or depression [32,36,56–59]. Therefore, understanding the role that location plays in fat talk interactions is an area of future research that would benefit from partnership with citizen sociolinguists and/or citizen scientists generally.

#### Citizen sociolinguistics data show fat talk occurs among and between men and women

Data collected by our research teams show that men do engage in discussions about appearance, both with other men and with women (either as initiator or respondent) (see Table 3). In contrast, the literature indicates that fat talk falls primarily in the domain of women, particularly young adult women [23, 60]. Of the 374 conversations that involved physical appearance, the majority were initiated by women (69%). Twenty-seven percent of such observations were initiated by men. Mixed-gender conversations were recorded 20% of the time. To our knowledge, this is a novel discovery. Only 3.5% of observations failed to document the gender of the initial speaker. These results indicate that citizen sociolinguists capture more gender-diverse data than do typical fat talk studies, which, by design, tend to focus exclusively on women’s talk with other women or men’s recognition of it [37, 56, 61–63].

**Table 3:** Frequency of appearance-related talk by gender.

Speaker 1 Gender	Speaker 2 Gender	Frequency (%) of Documented Appearance Talk
**Female**	Female	52
	Male	10
	Unknown/Unreported	7
**Male**	Female	10
	Male	17
	Unknown/Unreported	< 1

Interestingly, there was no difference by gender of the citizen sociolinguist observer on the odds of recording a male-male interaction (p-value > 0.05, Odds Ratio (OR) contained 1.0). Male citizen sociolinguists were, however, statistically less likely than women citizen sociolinguists to record women-women interactions (OR = 0.113-.691, B = .279, p-value = 0.006), and more likely to observe men-women (mixed) interactions (OR = 1.09–9.09, B = 3.155, p-value = 0.33).

Our findings may stem, in part, from the fact that the research teams had more women than men. Nonetheless, it is clear that fat talk can be initiated by men or women to engage women or men. An area of fruitful research would be to investigate the content and structure of fat talk among men, as it (1) happens consistently in public settings and (2) is a traditionally understudied area in social science research [56, 64].

#### Citizen sociolinguistics data show fat talk occurs among people of diverse ages

Results show that young adult, middle-aged, and older people all engage in fat talk (Table 4).

**Table 4:** Frequency of appearance-related talk by age category.

Speaker 1 Age	Speaker 2 Age	Frequency (%) of Documented Appearance Talk
**Young (18–30)**	Young	46
	Middle	6
	Older	1
**Middle (31–50)**	Young	15
	Middle	10
	Older	5
**Older (51+)**	Young	3
	Middle	2
	Older	2

Conversations involving physical appearance were overwhelmingly initiated by individuals who appeared to be age 30 or under (60%), with 46% of documented interactions being between two younger individuals. Thirty-two percent of observations were initiated by people estimated between the ages of 31–50 (middle aged), with 15% of observations documented as being made toward a younger interlocutor and 10% to another middle-aged individual. Just 7% were initiated by an individual in the older age category (age 51+), rarely toward other older individuals (2%). Only 2% of observations failed to have a documented age estimate for speaker 1. Like the data collected by research teams, the literature also tends to focus on fat talk among younger people. That said, the research teams did capture data from middle-aged and older people—a phenomenon rarely documented in the literature [57–58, but see 61 as an exception]. Notably, older citizen scientists were less likely to observe younger conversants (OR = 0.51–0.442, *B* = 1.50, p-value = 0.001).

The focus on high school and university students in previous fat talk investigations has given the impression that it is more common among young people [see 20–21, 23, 25). However, the data collected by research teams demonstrate that while it may be more frequent among younger adults, everyone, regardless of age, engages in it. Additionally, our data show that age status is not a barrier to initiating fat talk–young people can engage someone older and vice-versa. Therefore, a fruitful next step would be to investigate responses to fat talk across and among age categories, as this would reveal how the relative ages of interlocutors can have consequences for expressions of politeness and how variation in responses to self-deprecation impacts the function of fat talk among diverse speakers.

### Assessment of citizen sociolinguists’ inclusion and experiences

#### Citizen sociolinguists as informal science educators

All nine citizen sociolinguists who completed the post project questionnaire stated that understanding fat talk was important. When asked to explain why, three citizen sociolinguists made explicit reference to psychosocial impacts of such talk. For example, one noted that occurrences of fat talk have “a relationship w[ith] social and emotional well-being.” Others referenced the way fat talk speaks to broader social phenomena including “[p]ower [d]istribution and expected performance inside society.” Additional responses focused on the importance of learning how these forms of talk affect communication. All citizen sociolinguists were assessed by researchers on their teams as having attained competence in identifying and understanding the social functions of fat talk.

Moreover, 7 of the 9 participants reported discussing the project or the topic of fat talk with friends, relatives, or colleagues. For open-ended responses about project benefits, 4 of 9 citizen sociolinguists cited having a greater awareness of conversations taking place around them and/or greater self-awareness about fat talk. For example, one person stated becoming “more aware of those around me and how obsessed we are as a society with our body image” which made them more likely to discuss fat talk with friends, co-workers, and family. Another person specifically stated that they felt inclined to learn how to “shift this [fat talk] behavior” so that she could educate others. In this way, the citizen sociolinguists engaged in informal science education within local communities and personal networks. These opportunities allow for a fuller analysis of the potential forms and functions of fat talk beyond what can be learned from lab or controlled studies, contributing directly to scientific knowledge.

#### Citizen sociolinguists’ participation in the scientific process

In terms of confidence in data collection, citizen sociolinguists judged themselves highly confident in their ability to both recognize fat talk and document it using the standardized template. Skills acquired included the ability to recognize and discriminate fat talk utterances from other comments about body weight or size, indicating heightened awareness of verbal interactions made by themselves and others. The citizen sociolinguists also reported noticing more details about interlocutors as well as the contexts in which particular kinds of fat talk occurred. After only a few weeks, they were able to recognize the difference in kinds of fat talk, including self-disparaging talk versus disparaging talk about others (e.g., fat shaming). They reported less confidence in their ability to identify simple demographic information about the interlocutors (e.g., age category).

#### Drawbacks to citizen sociolinguistics for participants

Our close engagement with citizen sociolinguists allowed us to investigate the articulation of scaling up a project while also focusing on inclusion in real time via project reflections on the research project, with special attention to what could be done differently in the future. When asked about drawbacks of participation, three citizen sociolinguists stated that there were no drawbacks and three cited the time for documentation as a hindrance. One said “finding time” to fill out the documentation sheet was difficult. Three cited concerns about hand documenting the utterances quickly enough. For instance, one person said that she “hated handwriting” and wished she could “audio record into a voice recorder” the instances of fat talk she observed. When asked for any recommendations to strengthen the project moving forward, several citizen sociolinguists pointed to the use of the binder as “cumbersome” and “difficult to remember” to take it along when leaving the house. To this end, two respondents specifically recommended using an electronic form of data collection because it could be done on a smart phone which would allow for more discreet documentation while alleviating the need to carry a binder.

### Suggestions for methodological improvements

As with any proof-of-concept project there is always room for improvement. Several points of confusion arose during the weekly check-in meetings. Many of these were documentation issues including how to document utterances by single speakers or by more than two speakers, as well as how to cope with the multimodality of human interaction, in particular documenting nonverbal communication such as gesturing (e.g., patting one’s belly to signify feeling fat or full). These issues were easily corrected via updated protocols.

Citizen sociolinguists indicated that a digital form of data collection that allowed data entry via a phone or other smart device would be quicker, less tedious, and less conspicuous. Researchers also felt that electronic data collection would be more efficient because transcription would no longer be necessary, only more stringent quality control checks. Based on this feedback, piloting of digital fat talk data collection via smart phones is currently underway.

While the benefits of collaborating with citizen sociolinguists on fat talk research are clear, stronger measures to assess the benefits of project participation to citizen sociolinguists could be useful (see [65] for further discussion). Examples might include a standardized means to assess changes in skill proficiency, spatial awareness or understanding of sociolinguistic theory over the project duration as these would demonstrate the practical benefits of participation, an important retention tool [49]. This may be particularly useful for citizen social sciences (including sociolinguistics), given citizen scientists may view social science concepts as more socially-constructed than biophysical science concepts.

We identified several limitations of sociolinguistic data collection through citizen sociolinguistic methods. The first limitation comes from the citizen sociolinguists themselves and who they are as individuals. That is, the citizen sociolinguist research teams collected fat talk data from within their own social networks. Consequently, the documented fat talk sample includes age, gender, and location biases commensurate to the ages, genders, and locations of the citizen sociolinguist research teams and their networks. However, this may be advantageous in many ways, especially with regard to age. Many studies of language interaction are biased toward younger speakers, and citizen sociolinguistic methods can capture greater age diversity if the citizen sociolinguists themselves are also diverse in age.

Other limitations, as noted above, stem from the difficulty in asking lay researchers to identify classic social scientific identity markers like race/ethnicity, age, sex, education level, or income. These are categories of information which may be difficult or impossible for citizen sociolinguists to document. Additionally, while citizen sociolinguist research teams documented many instances of fat talk, we cannot know (or even estimate) how many utterances were made by the same person or people over time. Presumably they collected utterances from the same people on a routine basis; if one documents fat talk at the workplace, then the cast of characters is limited. For example, if the citizen sociolinguist’s workplace overwhelmingly employs women (or men), then fat talk data they collected may exhibit gender bias. A final limitation is the issue of assessing the frequency of fat talk. If frequency estimates of fat talk are desired, a more structured research design (addressing sampling and direct observation) would be appropriate, such as sending future citizen sociolinguists out at specific times to multiple locations. In this way, a sense of frequency over space/location could be ascertained.

Finally, research shows that projects benefit from different levels or scales of participation that allow citizen scientists with prior project experience or relevant empirical expertise to take on more responsibility or projects which accommodate variation in the time or energy that different individuals can invest [48,66]. This can foster motivation and sustain adherence to the project and its goals. While we did not have different levels of participation in this project, in the future seasoned citizen sociolinguists could have their roles elevated to team leader and could also be involved in recruitment or have higher level roles in project development.

## Conclusion and broader implications for the future of sociolinguistic research

Citizen sociolinguistics is an emerging field of scientific investigation that offers the potential of scaling up empirical examinations of everyday language use. It additionally offers the opportunity for inclusivity across multiple domains (languages, ages, genders, race/ethnicity, and residences) which are at the heart of everyday language interaction. The benefit of collaborating with citizen sociolinguists is that data collection moves organically with the researcher as s/he moves through day-to-day social environments. To date, there has been a pervasive bias in fat talk research in that it focuses on samples of convenience, usually drawing from undergraduate researcher populations or very specific/targeted groups (e.g., high school students) (e.g., [19, 26, 64]. While such work is deeply informative, it has limited interpretive power in broader contexts. In our study, citizen sociolinguistic research teams captured natural variation in fat talk that revealed greater sample diversity (e.g., influence of gender and age) and environmental contexts (e.g., how location affects fat talk) than has been documented in the past. We found data collected by citizen sociolinguists to be reliable and valid, mirroring that collected by the trained researchers [see 47]. More than reliable and valid data collection, however, the finding that citizen sociolinguists engaged in informal science education in their local communities suggests that this methodology can have effects far beyond the original research question. This finding demands that we ask the following question: Could citizen sociolinguists (and citizen scientists) become informal community science educators that bridge gaps in the realm of public (psychosocial) health outreach programs? It is an intriguing idea.

Beyond implications for the scope and variation of scientific output produced by the research teams, our study showed that citizen sociolinguistics can make unique contributions to the field of sociocultural linguistics. In our case, citizen sociolinguists contributed data and interpretations that broadened our understanding of fat talk in terms of who engages in it and where. The data produced by citizen sociolinguistic research teams confirmed that it is not just important for understanding psychosocial health, but that it is an interaction with clear sociolinguistic importance. We also suggest that this methodology could prove important for other areas of study, including the growing field of medical humanities [67]. The form of the interaction, like other similar interactions (e.g., compliments, apologies, requests, and so forth), is embedded in the perceived social distance and situated relationships of the interlocutors [e.g., 13, 55, 68]. These interactional dynamics expressed as patterns in conversational form not only reflect the relationships among interlocutors, but mediate broader perceptions about an individual’s perceived status as defined by social norms related to factors such as body, gender, or age. With more expansive and targeted studies of spontaneous, naturally-situated verbal interactions in the future, citizen sociolinguists can further advance our understandings of discursive interactions in ways that were previously impossible.

## Supporting information

S1 File Data collection sheet used by citizen scientists to capture overheard fat talk utterances. (PDF)

S2 File Spreadsheet of data used in this research. (XLSX)
