# Supplementary material for: Citizen sociolinguistics: A new method to understand fat talk
Source: PLoS One. 2019 May 29;14(5):e0217618. doi: 10.1371/journal.pone.0217618 (PMC6541281; doi:10.1371/journal.pone.0217618)
Supplement: S1 File — (PDF) [file pone.0217618.s001.pdf]

Site/Location: ________________ Researcher ID: _________

Date: ______________ Interaction # (of the day): _____

1. Write the comment that the first speaker (Speaker 1) says (as best as you can recall).

____

____

____

If there was a verbal response, complete questions 2-4. Otherwise, skip to question 5.

2. Write the response given by the second speaker (Speaker 2):

____

____

3. If Speaker 1 replies to Speaker 2, write it below:

____

____

4. If Speaker 2 replies again, write it below:

____

____

5. Nature of reply:

a. Redirect (response did not address comment)
b. No reply given (comment was met with silence)
c. Noticeable pause, then reply
d. Chuckle/laughter, then reply
e. Reply given without pause

6. Were you directly involved in the interaction? ___ Yes ___No

7. *If yes*, did you (check one): ____ Initiate the conversation (you were speaker 1)

____ Respond to a comment made by someone else (you were speaker 2)

8. Please provide the following information about the people conversing:

	Number of people interacting:		
	Relationship among people involved (if known): spouse, children, friends, sibling, other:		
	Location of interaction:		
Speaker 1	___ Lower Age<br>___ Upper Age	Confidence<br>– 1 2 3 4 5 6 7 8 9 +	Justification
	Sex: M or F		
	Race:		
Speaker 2	___ Lower Age<br>___ Upper Age	Confidence<br>– 1 2 3 4 5 6 7 8 9 +	Justification
	Sex: M or F		
	Race:		

9. Any other feedback that you feel is relevant to understanding this interaction? ___ Yes ___ No

If yes, please write this feedback on the back of this form.
